# Tracing the evolutionary origins of insect renal function

**DOI:** 10.1038/ncomms7800

**Published:** 2015-04-21

**Authors:** Kenneth A. Halberg, Selim Terhzaz, Pablo Cabrero, Shireen A. Davies, Julian A. T. Dow

**Affiliations:** 1 Institute of Molecular, Cell and Systems Biology, College of Medical, Veterinary and Life Sciences, University of Glasgow, Davidson Building, Glasgow G12 8QQ, UK; 2 August Krogh Centre, Department of Biology, University of Copenhagen, Universitetsparken 13, DK-2100 Copenhagen, Denmark

## Abstract

Knowledge on neuropeptide receptor systems is integral to understanding animal physiology. Yet, obtaining general insight into neuropeptide signalling in a clade as biodiverse as the insects is problematic. Here we apply fluorescent analogues of three key insect neuropeptides to map renal tissue architecture across systematically chosen representatives of the major insect Orders, to provide an unprecedented overview of insect renal function and control. In endopterygote insects, such as *Drosophila*, two distinct transporting cell types receive separate neuropeptide signals, whereas in the ancestral exopterygotes, a single, general cell type mediates all signals. Intriguingly, the largest insect Order Coleoptera (beetles) has evolved a unique approach, in which only a small fraction of cells are targets for neuropeptide action. In addition to demonstrating a universal utility of this technology, our results reveal not only a generality of signalling by the evolutionarily ancient neuropeptide families but also a clear functional separation of the types of cells that mediate the signal.

## Abstract (web summary)

The evolution of neuropeptide signalling in insects is poorly understood. Here the authors map renal tissue architecture in the major insect Orders, and show that while the ancient neuropeptide families are involved in signalling in nearly all species, there is functional variation in the cell types that mediate the signal.

## Graphical abstract

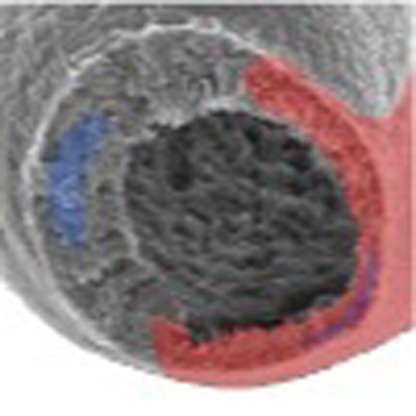

In insects, the regulation of complex homeostatic processes relies on the coordinated neurohemal release of small signalling molecules, neuropeptides, which bind to their cognate receptors in target tissues to elicit a physiological response. Neuropeptide systems are thus key mediators of intercellular communication and direct critical actions in morphogenesis, metabolism, reproduction, behaviour and renal function in insects^1^,^2^,^3^. The neuroendocrine control of fluid and ion homeostasis is particularly complex and is considered a physiological adaptation to a high surface area to volume ratio, which renders insects highly susceptible to osmotic stress^4^. In the genetic model *Drosophila melanogaster*, the renal (Malpighian) tubules are characterized by two main physiologically distinct secretory cell types—the principal cell (PC) and the secondary (stellate) cell (SC)—that receive multiple separate neuropeptide signals (Fig. 1a,b). However, whether this two-cell-type model and associated neuropeptide signalling is uniform across all insects is remarkably unexplored^4^.

In every insect species studied to date, the kinin neuropeptides have been shown to activate the chloride shunt conductance and water transport^5^,^6^,^7^,^8^, and in *D. melanogaster*, as well as the malaria and dengue vectors, *Anopheles gambiae* and *Aedes aegypti*, kinin receptor expression in Malpighian tubules (MTs) is confined to SCs^9^,^10^,^11^. By contrast, the capa and calcitonin-like (DH_31_) receptors have been shown to localize to PCs, where they act through independent cell signalling pathways to increase primary urine production^12^,^13^,^14^,^15^. Thus, while the kinin receptor is diagnostic of SC function, capa and DH_31_ receptors are markers of PC activity (Fig. 1b). Yet, there is limited morphological evidence for two secretory cell types outside the large Order of Diptera^4^, and so a single, ‘general-purpose' cell type is presumably the plesiomorphic condition in insects. It is plausible that two morphologically identical but functionally distinct (latently specialized) cell types are present; however, we currently lack the tools to distinguish between these different epithelial models in other insects. Indeed, addressing this problem is unusually complex, because insects—with an estimated 10–30 million extant species—make up >60% of known global animal biodiversity^16^, and so experimental sampling of even a fraction of this group would be prohibitively slow and expensive. Taming insect biodiversity therefore represents a grand challenge to modern physiology.

One approach could be to screen for orthologues of genes diagnostic of the SC identity and to localize their expression to specific cell types. For example, small cells positive for *tiptop* orthologue expression—a gene coding for a transcription factor implicated in SC differentiation in *Drosophila*—have been identified in members of Coleoptera and Orthoptera^17^,^18^, raising the possibility that previously undetected SCs could be widespread in insects. Nevertheless, this approach requires prior sequence information for the orthologues in every species of interest, for example to generate antibodies, or design *in situ* probes. Another SC-specific gene could be that encoding for the kinin receptor, but in spite of a well-conserved topographical structure, G protein-coupled receptors display low levels of sequence homology between insect clades^19^, and would thus require extensive work for each new species studied.

In contrast to their cognate receptors, the functional core of most insect neuropeptide families are evolutionarily highly conserved (Supplementary Tables 1–3), with identified neuropeptide-receptor pairs evident beyond the insect lineage, to crustaceans, ticks, tardigrades, nematodes and even snails^20^,^21^,^22^,^23^,^24^, indicating a deep orthology between neuropeptide systems in Protostomia^25^,^26^. Moreover, several neuropeptide families have been shown to cross-activate receptors from other species separated by hundreds of millions of years of evolution^12^,^27^,^28^,^29^,^30^. Accordingly, we rationalized that fluorescent analogues of archetypal insect neuropeptides, in combination with advanced bioimaging techniques, could be used to directly visualize ligand binding, and thus provide a rapid, sensitive and cost-effective assay for mapping renal tissue architecture across the insect biodiversity (Fig. 1c); an approach that has previously been used to study ligand–receptor interactions in live cell cultures^31^. Here we adapt and optimize this methodology for application on live intact tissue *ex vivo*, allowing us to rapidly identify sites of neuropeptide action (including the subcellular localization of binding within individual cells) in strategically selected representatives of every major insect Order—covering nearly 400 million years of evolution and >90% of insect biodiversity^32^—thus providing an unparalleled overview of the evolutionary origins of insect renal function.

## Results

### Proof of concept

To validate our methodology, we synthesized a *Drosophila* kinin (DK) analogue conjugated to a stable, high quantum-efficiency fluorophore (TMR-C_5_-maleimide 543 Bodipy dye; DK-F), which we initially tested in *Drosophila*. This approach allowed us to exploit the availability of existing tissue expression data as well as independent resources for identifying receptors and quantifying function in this powerful genetic model. A bioinformatic analysis (flyatlas.org^33^,^34^) on the spatial expression pattern of the *Drosophila* kinin receptor (DKR) revealed that the receptor is most highly expressed in the central nervous system (CNS) and epithelial tissues such as the hindgut and tubule of both larval and adult *Drosophila* (Supplementary Fig. 1a,b). Accordingly, these tissues were selected for study.

In MTs of *Drosophila*, DKR is expressed exclusively in SCs^9^. DK-F application showed a clear colocalization both with DKR immunoreactivity as well as with a GFP-tagged DKR-targeted transgene in SCs (Fig. 2a). The DK-F signal was displaced by excess unlabelled peptide in a ligand competition assay, thus confirming the specificity of binding (Fig. 2b). Indeed, quantifying signal intensity in optical sections of SCs revealed a distinct peak at their basolateral membranes, which was significantly reduced upon coapplication of DK (Fig. 2c). Moreover, time-lapse confocal microscopy demonstrated DK-F binding to basolateral membranes of SCs within seconds of peptide application, with the signal intensifying over time as the peptide appears to be internalized (Fig. 2d). These studies additionally indicated the presence of specialized membrane subdomains (lipid rafts), containing apparent receptor ‘hotspots' as evidenced by punctae with high-intensity fluorescence in SCs (Fig. 2d).

Binding of DK-F was further functionally validated in this system. Insect kinins and their cognate receptors are known to signal through intracellular calcium release^9^,^35^ and to stimulate MT fluid secretion^36^. Real-time cytosolic calcium measurements showed that both the labelled and unlabelled peptides induced a similar characteristic biphasic calcium release in SCs of *Drosophila* MTs (Fig. 2e), just as both peptides significantly stimulated fluid secretion (*P*<0.05, paired-samples *t*-test) compared with basal rates (Fig. 2f) in fluid secretion assays, with a biological activity that was not significantly different between the two peptides (*P*>0.05, unpaired two-sample *t*-test). Fluorophore labelling does therefore not affect the biological activity of the kinin neuropeptide. In addition to the tubule epithelium, we demonstrated a clear colocalization of receptor and DK-F signals in the adult hindgut and the larval CNS of *Drosophila*, as well as a functional stimulation of hindgut contraction frequency by both the labelled and unlabelled ligands (Supplementary Fig. 2). Collectively, these results show that DK-F consistently reports DKR localization across multiple tissues and that the labelled ligand is near equipotent regarding its biological activity compared with the native unlabelled peptide.

### Receptor-binding assay maps insect renal organization

To test the evolutionary scope of the two-cell-type model, we initially adapted the technique to cover other closely related members of Diptera, before expanding our experimental sampling to more distantly related species. In our sample coverage, particular emphasis was put on species with available genomes, to allow bioinformatic identification of neuropeptide/receptor gene pairs, as an independent validation of our receptor-binding assay. In addition, we sampled across the larger Orders, trying to choose species representative of the breadth of the Order. This approach allowed a compact sampling of nearly 400 million years of insect evolution^32^ and >90% of the total insect biodiversity (Supplementary Fig. 3).

Specific and displaceable DK-F binding to tubule basolateral membranes was observed across most insects sampled (Fig. 3), which is consistent with the ancestral origin of kinin/receptor gene pairs before the diversification of the insects^22^,^23^. Specific signals were detected both in the ‘advanced' endopterygote and ‘ancestral' exopterygote insect Orders; yet, surprisingly, kinin binding was not detected in any beetle (Coleoptera) species studied (Fig. 3f–i; Supplementary Fig. 3). These findings are independently corroborated by an *in silico* study, in which no kinin or kinin receptor gene could be identified in the *Tribolium* genome^37^. Another exception to the general pattern was observed in the aphid *Myzus persicae*, an exopterygote. Aphids have secondarily lost MTs, probably due to a highly specialized lifestyle; nevertheless, specific kinin binding was observed to localize entirely to circular muscle throughout the length of their alimentary canal (Fig. 3l), rather than epithelial cells, suggesting an exclusively myotropic action of kinins in Aphididae.

### Kinin binding predicts diuretic activity across species

A prerequisite of our approach is that kinin binding should predict a functional stimulation of primary urine production by insect renal epithelia. To test this, we developed and optimized the classical Ramsay fluid secretion assay^38^ for most of the species studied, and assayed against both labelled and unlabelled kinin. These experiments demonstrated that in every species specific kinin binding was observed, it was functionally active and consistently stimulated diuresis (Fig. 4; Supplementary Fig. 4).

### Phylogenetic distribution of the two-cell-type model

Integrating our experimental data and comprehensive bioinformatics on the kinin neuropeptide family with a consensus phylogeny of the investigated species of insects allowed us to gain insight into the evolution of insect renal function and control (Fig. 5). Analysing these data suggests that a dramatic change in insect renal tissue architecture arose ∼350 million years ago (mya) in the last common ancestor of the exopterygote and endopterygote Orders (blue triangle, Fig. 5), when the small SCs adopted kinin signalling to the exclusion of other cells thus leading to the partitioning of different transport functions into distinct cell types; the two-cell-type model. Further, our analyses suggest that a single ‘general-purpose' secretory cell type responsible for MT function is the plesiomorphic condition in insects, and that kinin signalling was secondary lost ∼260 mya in the largest insect Order, Coleoptera (purple triangle, Fig. 5). Taken together, our data suggest that kinin signalling is ancient relative to the insect lineage, is uniformly diuretic (and/or myotropic) in insects, but has been secondarily lost early in the coleopteran lineage.

### The unique neuroendocrine control of beetle renal function

**Figure 1:**
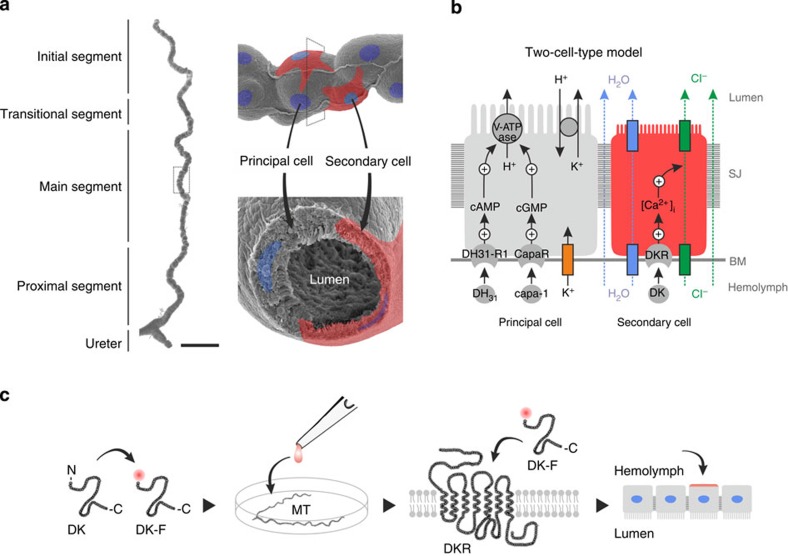
Mechanism and control of the *Drosophila* renal (Malpighian) tubule and principle of *ex vivo* receptor binding assay. (**a**) Spatial organization of the *Drosophila* tubule as evidenced by scanning electron microscopy, with superimposed illustrations of principal and secondary cells. Scale bar, 100 μm. (**b**) Classic two-cell-type model: fluid transport is energized by a vacuolar H^+^-ATPase (V-ATPase) located in the apical membrane of PCs (grey), which through a K^+^/H^+^ exchanger, drives net secretion of K^+^ into the lumen. Whereas basal levels of chloride flux take place through para- and/or trans cellular mechanisms (green arrows), kinin-stimulated rates of chloride transport are mediated by chloride channels in SCs (red)^8^. The chloride movement balances the net charge transfer, with osmotically obliged water (blue arrows) following through water channels in SCs and/or through paracellular routes. The DKR localizes uniquely to SCs, and controls, through stimulation with DK and subsequent intracellular calcium release, the chloride shunt conductance and thereby fluid transport. (**c**) A high quantum yield fluorophore (Bodipy TMR-C_5_-maleimide) is conjugated to the N-terminal region (non-functional end) via a cysteine linker to a synthetic analogue of the native neuropeptide (DK), hereby generating a fluorescently tagged neuropeptide (DK-F). The fluorescently tagged neuropeptide is applied to acutely dissected tissue (for example, MTs), where the labelled peptide binds to the endogenous receptor (DKR), hereby allowing direct visualization of neuropeptide-receptor interaction and subsequent identification of the cells that receive the neuroendocrine signal. BM, basement membrane; SJ, septate junction.

**Figure 2:**
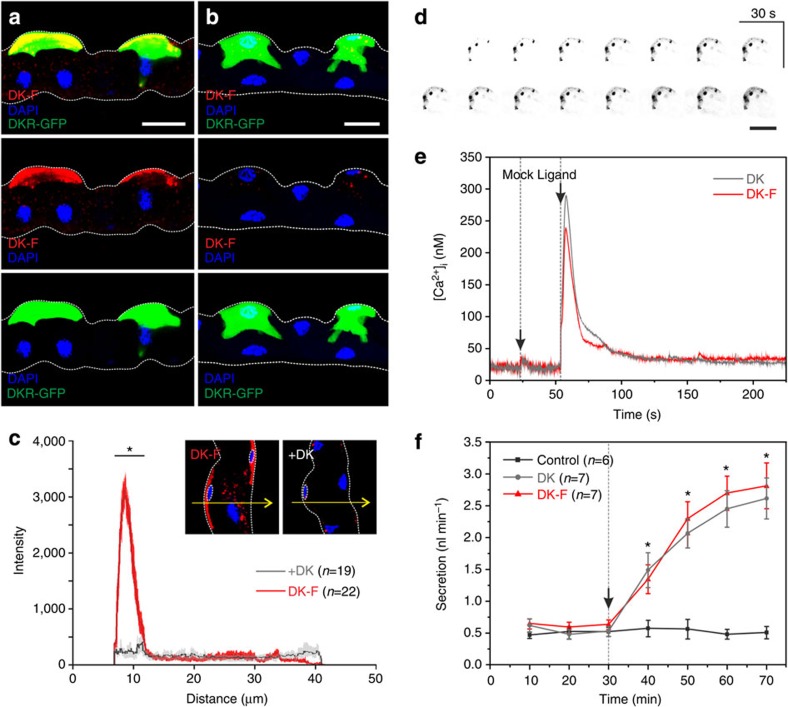
Fluorophore-labelled *Drosophila* kinin reports receptor localization in renal (Malpighian) tubules of *Drosophila*. (**a**) Application of DK-F (10^−7^ M) to tubules in which SCs are marked by DKR-GFP^48^ confirmed that the peptide co-localizes with DKRs on SCs. (**b**) Competitive inhibition with DK (10^−5^ M) almost fully abolished the fluorescent signal. (**a**,**b**) Maximum projections of confocal z-series. Scale bar, 25 μm. (**c**) Kinetic inhibition significantly reduced (*P<0.05, unpaired, two-sample *t*-test) the fluorescent intensity in SCs. The average intensity profile±s.e.m. of *n*=22 SCs from DK-F treatments showed a distinct peak for the basolateral membrane of SCs, which was not present in the average intensity profile±s.e.m. of *n*=19 SCs from+DK treatments. Arrows exemplify the x-y position of intensity traces made through optical sections (thickness: 10 μm) of individual SCs, as identified by smaller nuclei (stippled circle). (**d**) Time-lapse sequence of an individual SC from wild-type (Canton S) tubules following application of DK-F (10^−7^ M). Kinin binding can be visualized almost immediately, but increases in intensity over time as the receptor population becomes saturated and the peptide becomes internalized. Putative receptor ‘hotspots' are visible as punctae. Images are grey scaled and inverse coloured to increase signal clarity. Scale bar, 25 μm. (**e**) DK-F and DK stimulated calcium release following peptide application, indicates that the biological activity of DK is unaffected by fluorophore labelling, as evidenced by a very similar biphasic response. Black arrows indicate time of injection with either Schneider's medium (mock) or peptide (ligand). The traces shown are representative of *n*=3 separate experiments. (**f**) DK-F and DK significantly stimulate (*P<0.05, paired-samples *t*-test) fluid secretion rates compared with basal conditions in MTs (*n*=6–7) of *Drosophila*. Black arrow indicates time of peptide application. Values are expressed as mean±s.e.m.

In addition to DK-F, we generated two other key diuretic neuropeptide analogues (*Drosophila* capa-1-F and DH_31_-F) and validated them using a similar approach. The spatial expression patterns of the corresponding neuropeptide receptors (CapaR and DH_31_R-1) revealed that they are most highly expressed in the *Drosophila* adult tubules (Supplementary Fig. 1c–f), where they are known to localize to the PC^13^,^15^ (Fig. 1b). Similar to the labelled kinin analogue, specific and displaceable capa-1-F and DH_31_-F binding to PC basolateral membranes was observed across a wide phylogenetic sampling (Fig. 6; Supplementary Figs 5 and 6), and in every instance where we were able to perform a fluid secretion assay, they were demonstrated to be biologically active (Supplementary Figs 7 and 8). These data thus suggest that this methodology has universal utility in comparative endocrinology and this can be a powerful tool in unmasking sites of neuropeptide action, particularly in non-model species. Consistent with their known expression patterns in dipterans^13^,^39^, both capa and DH_31_ receptors localize exclusively to a PC type across the endopterygote insect Orders, but was more generally localized in the exopterygote *Gryllus assimilis* (Supplementary Figs 5g and 6g); neither capa-1-F nor DH_31_-F binding could be visualized along the length of the alimentary canal in the aphid *Myzus percicae*. These observations support the conclusion that the two-cell-type model for renal function and control is a derived trait, which evolved early on in the radiation of the endopterygote insects. Intriguingly, both capa-1-F and DH_31_-F reactivity were observed to colocalize to a small population of ‘inverse' secondary cells (iSC) in every beetle species studied (Supplementary Figs 5d–f and 6d–f), indicating that only a small fraction of cells receive the known neuropeptide signals in Coleoptera (Fig. 6). These iSC cannot be distinguished based on nucleus size or cellular morphology, indicating that they do not correspond to the small cells observed to express an orthologue of *tiptop* in *Tribolium castaneum*^17^. This suggests that additional molecular mechanisms are involved in cell-type diversification in Coleoptera^18^. Moreover, the capa-1 neuropeptide, which is universally diuretic in all other species tested, acts as an antidiuretic factor in MTs of *Tenebrio molitor* (Fig. 6; Supplementary Fig. 7; ref. 40), which implies that the downstream effects of capa signalling has been secondarily modified in beetles. Capa signalling was previously demonstrated to be antidiuretic in the hemipteran vector of Chagas disease *Rhodnius prolixus*^41^, indicating that although systematic co-evolution between neuropeptides and receptors is evident^26^, the physiological effects of receptor activation are subject to diversification, and has occurred multiple times throughout insect evolution.

## Discussion

**Figure 3:**
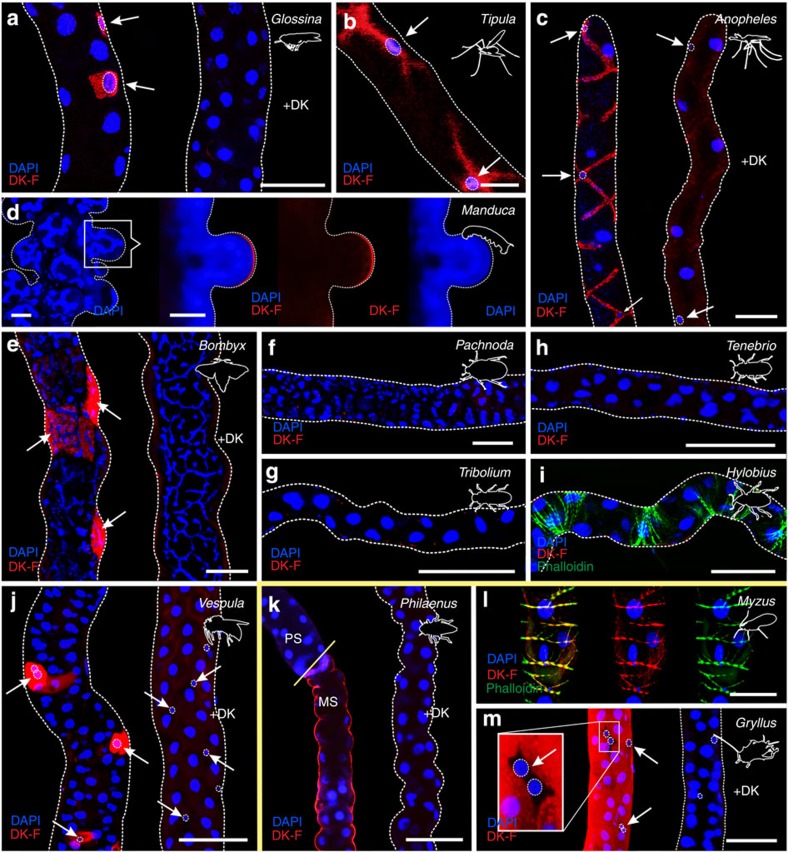
The receptor-binding assay is a tool to probe insect biodiversity. Application of DK-F (10^−7^ M) to insect MTs maps kinin receptors to a distinct cell type (arrows) in all endopterygote insects, such as (**a**) *G. morsitans* (Diptera), (**b**) *T. oleracea* (Diptera), (**c**) *A. gambiae* (Diptera), (**d**) *M. sexta* (Lepidoptera), (**e**) *B. mori* (Lepidoptera) and (**j**) *V. vulgaris* (Hymenoptera). In contrast, no staining was observed in tubules of the coleopterans (**f**) *P. marginata*, (**g**) *T. castaneum*, (**h**) *T. molitor* or (**i**) *Hylobios abietis*. In the exopterygote species, kinin receptors localize throughout the length of the tubule, as in (**k**) *P. spumarius* and (**m**) *G. assimilis*, or to circular muscle cells like in (**l**) *M. persicae* (secondarily lost MTs). Notably, the MT anatomy of *M. sexta* larvae is characterized by dome-like structures protruding throughout the length of the tubule, with each ‘dome' constituting a single cell containing a highly branched nucleus. Some of these ‘domes' demonstrated kinin-reactivity reminiscent of the leptophragmata cells in the cryptonephridial system of *T. molitor*^17^. The image shown represents an optical section made through one of these ‘domes' to emphasize basolateral localization of the DK-F signal. In all species tested, excess unlabelled peptide (+DK) greatly reduced the fluorescent signal. Yellow line separates the endopterygote and exopterygote species tested. PS, proximal segment; MS, main segment. Scale bar, 25 μm in (**a**–**c**), (**j**) and (**l**). Scale bar, 50 μm in (**d**–**i**), (**k**) and (**m**).

**Figure 4:**
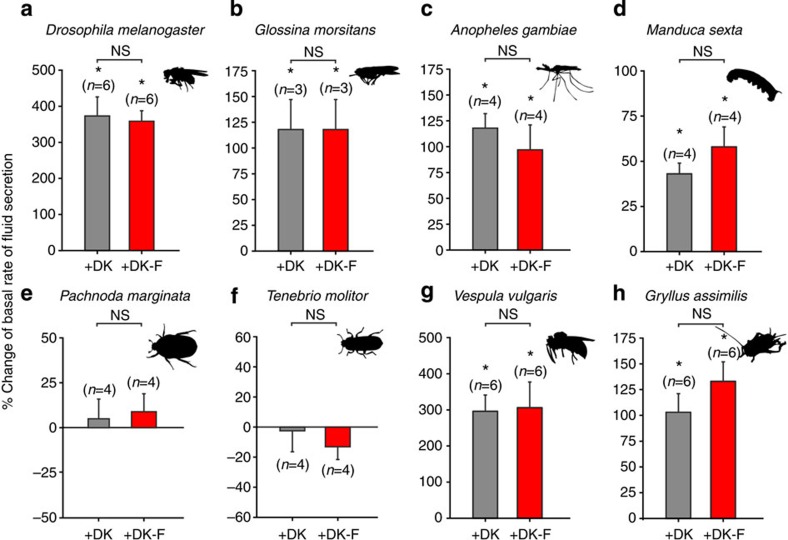
*Drosophila* kinin binding predicts diuretic activity across species. Percent change of basal rates of fluid secretion following stimulation with DK-F or DK on MTs from (**a**) *D. melanogaster* (**b**) *G. morsitans*, (**c**) *A. gambiae*, (**d**) *M. sexta*, (**e**) *P. marginata*, (**f**) *T. molitor*, (**g**) *V. vulgaris* and (**h**) *G. assimilis*. Values are expressed as mean±s.e.m. Significant difference from basal rates of fluid secretion immediately before peptide application compared with 30 min after stimulation was tested using a paired-samples *t*-test, while an unpaired two-sample *t*-test was used to test differences in secretory response between DK-F and DK treatments. In both cases, a significance level of *P<0.05 was taken as the critical value. NS, not significant.

**Figure 5:**
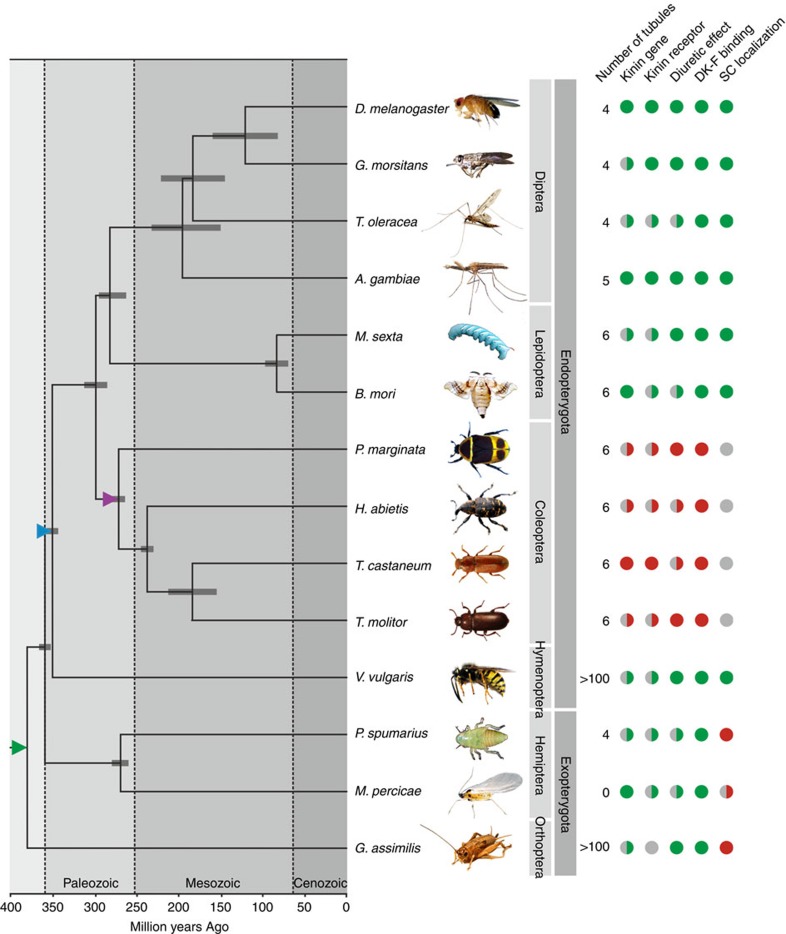
Phylogenetic distribution of the two-cell-type model for insect renal function. Consensus insect phylogeny of the insect species used in our study with superimposed character matrix. A full green circle denotes a positive, while a full red circle indicates a negative, for each category for each species. By contrast, a half green or red circle indicates that, for at least one member of that insect group, a positive or negative has been experimentally confirmed. A grey circle implies that data is not available. A coloured triangle indicates a key event in the evolution of insect renal function and control: Green triangle, SCs arose; blue triangle, SCs adopted kinin signalling; purple triangle, kinin-signalling secondary lost. The phylogenetic relationships and error bars reflecting 95% confidence intervals of the estimated divergence times for the branching nodes were adopted from refs 32, 46, 63, 64.

**Figure 6:**
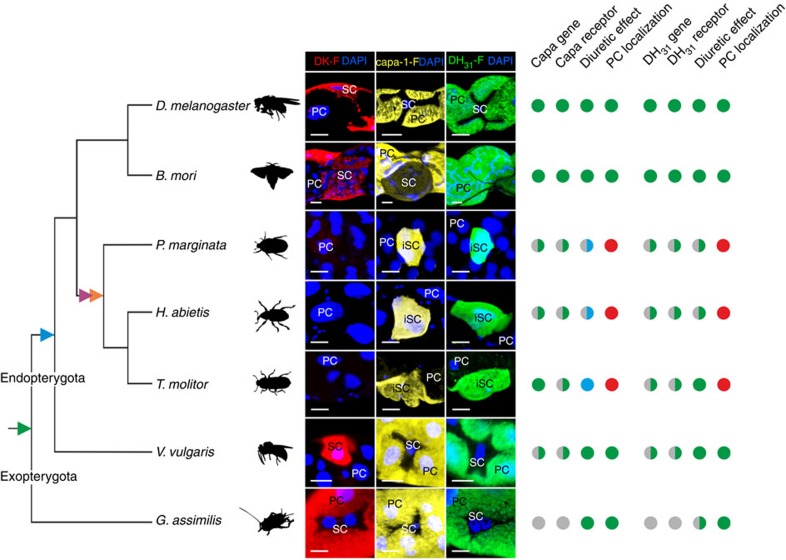
The unique neuroendocrine control of beetle renal (Malpighian) tubules. Consensus insect phylogeny (based on refs 32, 46) of the insect species in which all three neuropeptide analogues have been tested with superimposed character matrix. Images depict DK-F, capa-1-F and DH_31_-F reactive cells in MTs of phylogenetically diverse insect species, and the identification of PCs, SCs and iSCs. A full green circle denotes a positive, while a full red circle represents a negative, for each category for each species. A full blue circle indicates an antidiuretic response. By contrast, a half green, blue or red circle indicates that, for at least one member of that insect group, a positive or negative has been experimentally confirmed. A grey circle implies that data is not available. A coloured triangle indicates a key event in the evolution of insect renal function and control: green triangle, SCs arose; blue triangle, SCs adopted kinin signalling; purple triangle, kinin-signalling secondary lost; orange triangle, the iSC arose. Scale bar, 10 μm.

In this study, we introduced a novel tool to comparative endocrinology—that may easily be translated to other animal systems—to investigate the evolutionary origins of insect renal function and control, by systematically mapping renal tissue architecture across the insect biodiversity. Historically, insect renal function was described in terms of a single cell type; however, it has since emerged that kinin reactivity, chloride shunt conductance and water transport are entirely associated with the secondary cells in *Drosophila* and perhaps mosquitos^8^,^9^,^10^,^11^. Although MT structure and function have been studied extensively, secondary secretory cells have not been documented outside the large Order of Diptera, yet our receptor-binding assay unmasked their presence throughout the Endopterygota (Fig. 3). By contrast, the ancestral Exopterygota show uniform DK-F, capa-1-F and DH_31_-F reactivity along the renal epithelium, which indicates the lack of a secondary secretory cell type (Fig. 3; Supplementary Figs 5 and 6). So, while kinin signalling evolved before the radiation of the insects^20^,^21^,^22^,^23^, only the endopterygotes appear to possess a unique, specialized cell type that mediates the translation of the kinin signal into a secretory response (blue triangle, Figs 5 and 6). The physiological advantages associated with a partitioning of transport functions into two distinct secretory cell types might be to allow the uniquely high secretion rates observed in insects such as *Drosophila*, as well as to offer additional mechanisms of epithelial control critical to maintaining fluid homeostasis. Indeed, this functional innovation might have been a contributory factor to the evolutionary success of the endopterygote insects (Supplementary Fig. 3).

SCs have previously been documented in MTs of ancestral exopterygote insects^42^,^43^, and small cells expressing an orthologue of *tiptop* have been observed in both a beetle (*T. castaneum*) and a cricket (*Gryllus bimaculatus*) species^17^,^18^. These findings indicate that small SC evolved early in insect evolution (green triangle, Figs 5 and 6), and are likely present in all extant species. However, our data suggest that these cells are not sites of neuroendocrine activity (Figs 3 and 6; Supplementary Figs 5 and 6). This implies that *tiptop* expression and kinin signalling, previously considered intrinsically linked, are intertwined yet separable. Beetles lack kinin signalling altogether (Fig. 3f–i)^37^, and the cells identified in *G. bimaculatus* closely resemble those devoid of neuropeptide binding in our study (Figs 3m and 6). Accordingly, SC are present across the insects, yet perform a fundamentally different role in exopterygotes compared with endopterygotes, such as maintaining stemness^44^,^45^, regulating mucous production and/or carrying out unknown transport functions^42^.

Using a panel of archetypal fluorophore-labelled neuropeptides, we generated an authoritative overview of insect renal function and control (Figs 5 and 6). Surprisingly, these results revealed that coleopteran renal tissue architecture is separate from that of all other endopterygote insects, in that kinin signalling is secondarily lost, and that capa-1 and DH_31_ signalling is confined to a subpopulation of iSC (Figs 3 and 6; Supplementary Figs 5 and 6). Data mining the genome of *T. castaneum* (Coleoptera) further revealed that other signalling systems stereotypically involved in diuresis are greatly expanded^30^, emphasizing the fact that Coleoptera—the largest insect Order comprising >30% of the total insect biodiversity^46^—regulate fluid and ion homeostasis by profoundly different mechanisms than other insects.

## Methods

### Peptide synthesis

*Drosophila* kinin was selected as the archetypal peptide for our study because it is amongst the longest kinin sequences known (Supplementary Table 1). Therefore, an N-terminal label would be less likely to interfere with binding of the conserved C-terminal, which is necessary to confer biological activity. *Drosophila* kinin (both with and without an N-terminal cysteine) was synthesized by Cambridge Peptides (Birmingham, UK), and the modified peptide was subsequently coupled to TMR-C_5_-maleimide Bodipy dye (BioRad, CA, US), to make fluorescent TMR-C_5_-maleimide-CNSVVLGKKQRFHSWGamide (DK-F). The final working concentration was adjusted according to peptide purity (95.2 and 98.6%, respectively). The same rationale was used for selecting *Drosophila* capa-1 and DH_31_ (Supplementary Tables 2 and 3) as archetypal peptides of their respective neuropeptide families, which led to the generation of native (capa-1; GANMGLYAFPRVamide, purity 95.1%) and labelled capa-1 (capa-1-F; TMR-C_5_-maleimide-CGANMGLYAFPRVamide, purity 94.8%) and to native (DH_31_; TVDFGLARGYSGTQEAKHRMGLAAANFAGGPamide, purity 94.0%) and labelled DH_31_ (DH_31_-F; Alexa488-C_5_-maleimide-CTVDFGLARGYSGTQEAKHRMGLAAANFAGGPamide, purity 92.5%). The choice of fluorophores was based on their photophysical properties (for example, quantum yield and fluorescence lifetime) as well as their complementarity to our optical set-up (for example, lasers and filter sets).

### Animal models

*D. melanogaster* (Diptera): all fly lines were cultured on standard medium over a 12:12 h photoperiod at 45–55% humidity at 22 or 26 °C. Only ≥7-day-old flies were used for experimentation. The following strains were used: Canton S (wild type, WT), c724-Gal4 (ref. 47), UAS-CD8-GFP; DKR protein trap (FlyTrap YD0927 (ref. 48)) and c724-Gal4; UAS-GFP::aequorin^49^. *Glossina morsitans* (Diptera): parasite-free, unfed tsetse flies were a kind gift of Mr Craig Lapsley, University of Glasgow. Three- to five-day-old flies were used. *Tipula oleracea* (Diptera): adult specimens were wild caught in Glasgow during May–August 2013 and were used immediately for experimentation. *A. gambiae* (Diptera): mosquito larvae were a kind gift from Dr Lisa Ranford-Cartwright, University of Glasgow. Larvae were reared on fish food at 22 °C, while eclosing adults were maintained on 5% glucose (w/v) at 26 °C. Both larvae and adults were used for mapping kinin receptor localization; however, only tubules of adult females were used in fluid secretion assays. *Manduca sexta* (Lepidoptera): specimens of *M. sexta* were purchased as fourth instar larvae from University of Bath and reared as described previously^50^. Experiments were performed on late fifth instar larvae. *Bombyx mori* (Lepidoptera): silk moth larvae were obtained commercially and reared according to standard procedures (http://www.silkwormstore.co.uk/). *Pachnoda marginata* (Coleoptera): larvae were acquired commercially (http://livefoods.co.uk) and reared in moist soil and leaf litter at 26 °C. Both larvae and adults were fed on fruit and water *ad libitum*. *Hylobius abietis* (Coleoptera): specimens were wild caught in the south of England and were a kind gift of Dr Daegan Inward, Forest Research, UK. *T. castaneum* (Coleoptera): stock (San Bernadino) of the red flour beetle was a kind gift from Dr Gregor Bucher, University of Göttingen. Both larvae and adults were kept in whole meal organic flour supplemented with 5% dry yeast at 26 °C until experimentation. *T. molitor* (Coleoptera): larvae were obtained commercially from http://livefoods.co.uk/ and kept on bran *ad libitum* until experimentation. Potato slices were occasionally introduced to add moisture. Adults were kept under identical conditions. *Vespula vulgaris* (Hymenoptera): workers of *V. vulgaris* were wild caught in Glasgow during August 2013 and maintained on 5% glucose (w/v) at 18 °C for a maximum of 2 days before being used for experiments. *Philaenus spumarius* (Hemiptera): nymphs were collected around the campus of University of Glasgow during July–August 2013 and used immediately for experimentation. *M. persicae* (Hemiptera): stock of *M. persicae* was a kind gift of Brian Fenton, The James Hutton Institute, UK. The aphids were cultured on tomato plants, and only winged adults were used. *G. assimilis* (Orthoptera): adults were acquired commercially (http://livefoods.co.uk/) and were kept on bran, fruit and water *ad libitum*.

### Immunocytochemistry

Adult hindguts and third instar larval CNSs were dissected from *Drosophila* and subsequently fixed in 4% paraformaldehyde in PBS for 30 min. Following several washes in PBS, the tissues were incubated in 10% goat serum and 0.2% Triton X-100 (blocking buffer) for 2 h, followed by incubation with rabbit polyclonal anti-DKR primary antibody^9^ (dilution 1:500) in blocking buffer overnight at 4 °C. Samples were then washed repeatedly in PBS before incubation in 4′,6-diamidino-2-phenylindole (DAPI, 1 μg ml^−1^; Invitrogen, CA, USA), Rhodamine/Alexa-633 coupled phalloidin (Invitrogen; dilution 1:100) and/or Alexa Fluor 488 goat-anti-rabbit antibody (Invitrogen; dilution 1:250) in blocking buffer overnight at 4 °C. After extensive washes in PBS, the tissues were mounted on poly-L-lysine (0.1% w/v in H_2_O, Sigma-Aldrich, MO, US) covered 35 mm glass bottom dishes (MatTek Corporation, MA, USA) in Vectashield (Vector Laboratories Inc., CA, USA) and image acquisition was performed on an inverted Zeiss LSM 510 Meta confocal microscope (Zeiss, Oberkochen, Germany).

### *Ex vivo* receptor-binding assay

For the *ex vivo* receptor-binding assay, appropriate tissues were carefully dissected from cold anesthetized animals of each species, under a dedicated insect saline (Supplementary Table 4), and then mounted on poly-L-lysine-covered 35 mm glass bottom dishes. Next, the tissues were set-up in a matched-pair protocol, in which one batch was incubated in the appropriate insect saline added the labelled neuropeptide analogue (10^−7^ M) and DAPI (1 μg ml^−1^), while the other was incubated in just DAPI; the latter batch was used to adjust baseline filter and exposure settings to minimize autofluorescence during image acquisition. Images were subsequently recorded on an inverted confocal microscope (Zeiss LSM 510 Meta) or an epifluorescent microscope (Ziess Axioskop II) equipped with a high-resolution microscopy camera (Zeiss AxioCam MR) using baseline filter and exposure settings. A concentration of 10^−7^ M of the peptide analogues was chosen for this and subsequent assays, as this concentration has been shown to be the minimal concentration needed to produce a saturated receptor response^9^,^36^, thus optimizing the conditions for optical detection of ligand–receptor complexes.

As the formation of ligand–receptor complexes is a reversible process (receptors bind their ligands non-covalently), we performed a ligand competition assay to validate the binding specificity of the labelled neuropeptides. The competitive binding equilibria can be formalized as:


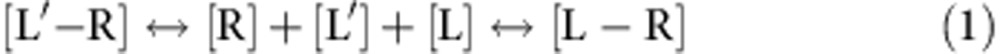


where R is the receptor, L′ is the labelled peptide and L is the unlabelled ligand^51^. According to equation (1), addition of L reduces [L′–R] in direct proportion to [L]. Therefore, an inhibition of fluorescent signal (that is, competitive displacement of the labelled ligand) under identical microscope settings, following coapplication of the labelled (10^−7^ M) and unlabelled (10^−5^ M) ligands, was taken as an indication of binding specificity. Further, to quantify the resulting reduction in fluorescent signal, intensity profiles from optical sections (10 μm) of multiple SCs (identified by smaller nuclei) from experimental and competitive inhibition treatments were generated. The intensity profiles were produced using the existing Zeiss LSM 510 Meta confocal software, and an average intensity trace±s.e.m. of *n*=19–22 SCs for both treatments was depicted as a function of distance in *x*–*y* position.

In addition, time-lapse experiments were set-up to investigate the time frame of DK-F binding to kinin receptors on individual SC in adult *Drosophila* MTs. Images were collected every 30 s following the application of DK-F (10^−7^ M) using the time series function of the Zeiss LSM 510 Meta confocal software. Data were exported as individual tiff-files for subsequent analysis.

### Hindgut contraction assay

A hindgut contraction assay was adapted from published techniques^52^,^53^,^54^,^55^. The entire alimentary canal was dissected from cold anesthetized adult WT *Drosophila* and preincubated in a freshly prepared mixture of Schneider's medium (Invitrogen, Carlsbad, CA, USA) and *Drosophila* saline (1:1, v/v) for 20 min, before being isolated in drops of the corresponding mixture under liquid paraffin. Each end of the alimentary canal was secured by minute metal pins in opposite ends of the droplet, thus applying a small but constant tension to the tissue. Recordings were made on a Sony NEX-C3 HD camera (Sony, Tokyo, Japan) mounted on a Leica WILD M3C compound microscope (Leica, Solms, Germany), with initial recordings of 2 min under basal conditions followed by 2 min after peptide application for each hindgut. The number of hindgut contractions before and after addition of the peptides was then visually counted, and the contractile response was presented as percent change in contraction frequency, with each hindgut serving as its own control.

### Ramsay fluid secretion assay

Fluid secretions were measured according to standard methods^56^. Intact MTs were dissected from native tissue and set-up as *in vitro* preparations by isolating them in drops of a freshly prepared mixture of Schneider's medium and insect saline (1:1, v/v) under water-saturated liquid paraffin, with one end being wrapped around a metal pin and the other end submerged in the saline drop. Secreted fluid would then accumulate as a discrete droplet from the common ureter or a small hole introduced midway between the drop and the pin. The tubules were left to secrete for ∼30 min, with non-secreting tubules being replaced if necessary, to produce a set of 10–20 working tubules. A drop of secreted fluid was subsequently collected at appropriate intervals and the diameter was measured using an eyepiece graticule. The volume of each droplet was calculated as (4/3)πr^3^, where r is the radius of the droplet, and secretion rates plotted against time. Secretion was measured under basal conditions in order to establish a steady rate of secretion, before stimulation with the labelled or unlabelled peptides, with an increase in fluid secretion rate being taken as an indication of a diuretic effect. For each species, the above-mentioned protocol was modified to accommodate differences in anatomy, physiology and size of the tubules. For *M. sexta*, *G. morsitans*, *P. marginata* and *T. molitor*, both cut ends of each tubule were pulled out into the paraffin and wrapped around metal pins, thus creating a pseudo ligature at each end, and a small hole was introduced as described above^28^,^39^,^40^. For *A. gambiae*, *V. vulgaris and G. assimilis* the cut (proximal) end of the tubule was wrapped around a metal pin with the intact distal end bathed in the droplet, and a small hole was made^57^,^58^,^59^. The insect salines used for acute dissections and functional assays were experimentally adjusted for each species, and are listed in Supplementary Table 4.

### Real-time measurements of intracellular calcium release

Cytosolic calcium measurements were performed as previously described^35^,^49^. In brief, flies were anesthetized on ice, and tubules expressing UAS-GFP::aequorin driven by c724-GAL4 (SC specific^47^) were dissected. For each sample, 20 pairs of live intact tubules were transferred to 5-ml Röhren tubes (Sarstedt AG & Co., Nümbrecht, Germany) in 175 μl Schneider's medium and subsequently incubated in the dark with 2 μl coelenterazine in ethanol (final concentration of 2.5 μM) for 3 h to reconstitute active aequorin. Real-time luminescence was measured on a Berthold Lumat LB 9507 luminometer (Berthold Technologies, Bad Wildbad, Germany). A stable baseline was established before both mock and subsequent injection with the labelled or unlabelled peptides (final concentration 10^−7^ M), and the luminescence was measured in the ensuing period. After the experiment, undischarged aequorin was measured by permeabilizing the cells with 300 μl lysis buffer (1% v/v, Triton X-100+0.1 M CaCl_2_). Real-time calcium concentrations throughout the experiments were then back calculated with an in-house PERL routine^35^.

### Scanning electron microscopy

Scanning electron microscopy was performed to visualize MT structure^60^,^61^,^62^. Briefly, MTs were dissected from 7-day-old flies (males and females) under Schneider's insect medium and were subsequently fixed in 2.5% glutaraldehyde in 0.1 M cacodylate buffer (pH 7.4) for 90 min. The tissue was then rinsed repeatedly in ddH_2_O before being dehydrated through a graded series of ethanol, and critical point dried using an Autosamdri-815 critical point dryer (Tousimis Research Corporation, Maryland, USA). Next, the tubules were transferred to aluminium stubs and coated with platinum (70 s∼12 nm thickness) in a JEOL JFC-2300HR high-resolution fine coater (Jeol, Tokyo, Japan) before being examined in a JEOL JSM-6335-F scanning electron microscope (Jeol, Tokyo, Japan).

### Statistics

The statistical analyses were performed using the data analysis software OriginPro 8.5 (OriginLab, MA, USA). All data are plotted as mean±s.e.m. Significant difference in hindgut contraction frequency and tubule secretory response following peptide application was tested using a paired-samples *t*-test, while an unpaired, two-sample *t*-test was used to test differences in fluorescent intensity between experimental and competitive inhibition treatments, as well as differences between DK and DK-F treatments. In all tests, *P*<0.05 (two tailed) was taken as the critical value.

## Author contributions

J.A.T.D. and K.A.H. conceived and designed the study with input from S.A.D. K.A.H. performed all experiments with assistance from S.T. and P.C. All authors contributed to the interpretation and analyses of data. K.A.H. and J.A.T.D. wrote the paper with inputs from all authors.

## Additional information

**How to cite this article:** Halberg, K. A. *et al*. Tracing the evolutionary origins of insect renal function. *Nat. Commun*. 6:6800 doi: 10.1038/ncomms7800 (2015).

## Supplementary Material

Supplementary Information Supplementary Figures 1-8, Supplementary Tables 1-4 and Supplementary References
