# Supplementary material for: Tracing the evolutionary origins of insect renal function
Source: Nat Commun. 2015 Apr 21;6:6800. doi: 10.1038/ncomms7800 (PMC4410669; doi:10.1038/ncomms7800)
Supplement: Supplementary Information — Supplementary Figures 1-8, Supplementary Tables 1-4 and Supplementary References [file ncomms7800-s1.pdf]

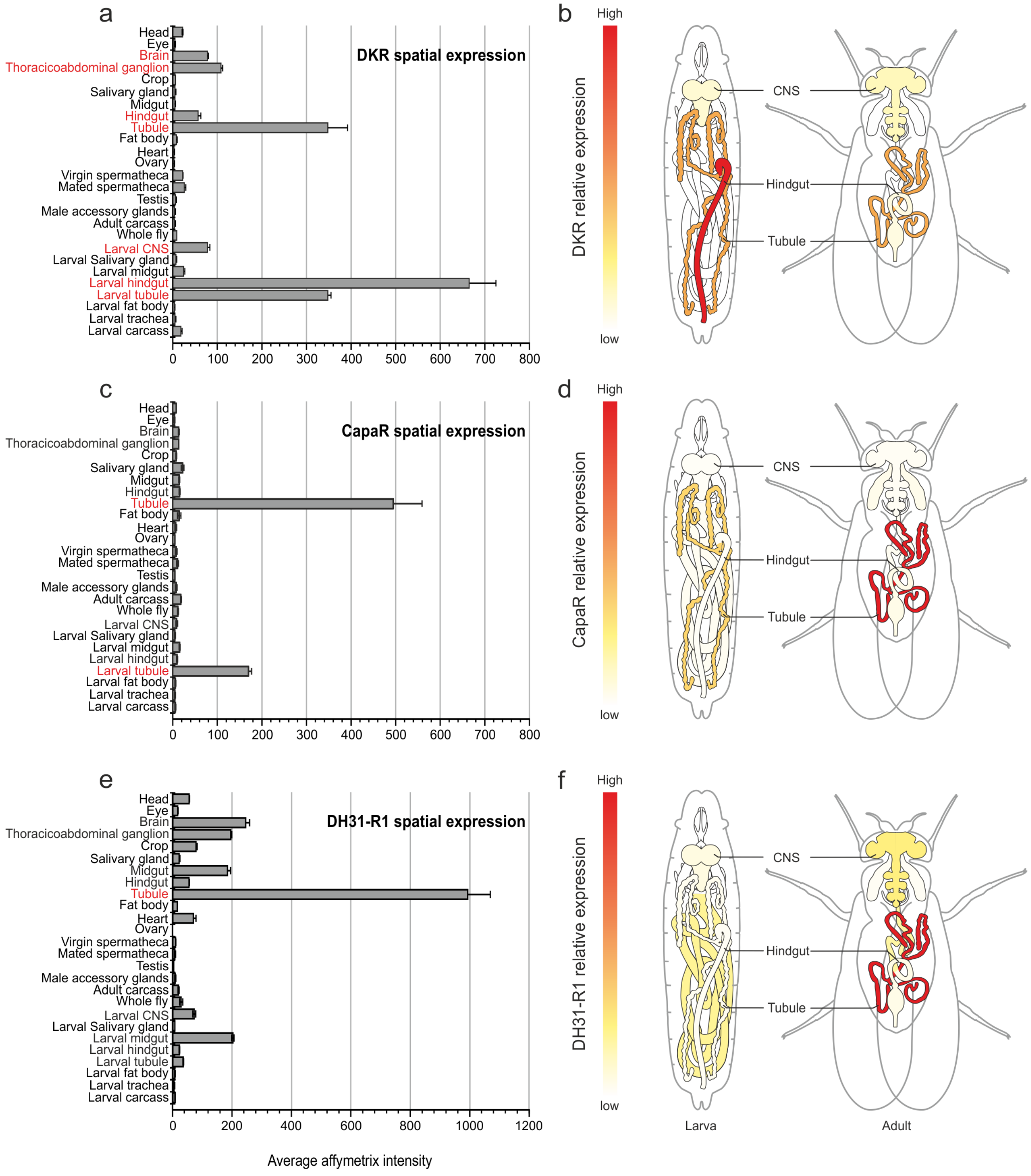

Supplementary Figure 1 | Tissue and developmental specific expression profile of the *Drosophila* kinin (DKR), capa (CapaR) and DH$_{31}$ (DH31-R1) receptors. (**a**, **c**, **e**) Mean normalized Affymetrix signal ± s.e.m. (n=4 tissue samples) showing the DKR, CapaR and DH31-R1 spatial expression pattern across major tissues from both larval and adult *Drosophila* (flyatlas.org[1, 2]). Tissue names in red indicate tissues in which the receptors are most highly enriched compared to whole fly. (**b**, **d**, **f**) Overview of *Drosophila* anatomy with superimposed heat maps of the spatial expression pattern of the receptors, highlighting the tissues in which the highest levels of the receptors are expressed. Based on these heat maps, the CNS, hindgut and MTs of both larva and adult *Drosophila* were identified as candidate tissues for experimental validation of our *ex vivo* receptor-binding assay (DK-F and DKR interaction), while adult tubules were subsequently used for demonstrating specific binding of CapaR and DH31-R1 with their respective fluorescent ligands.

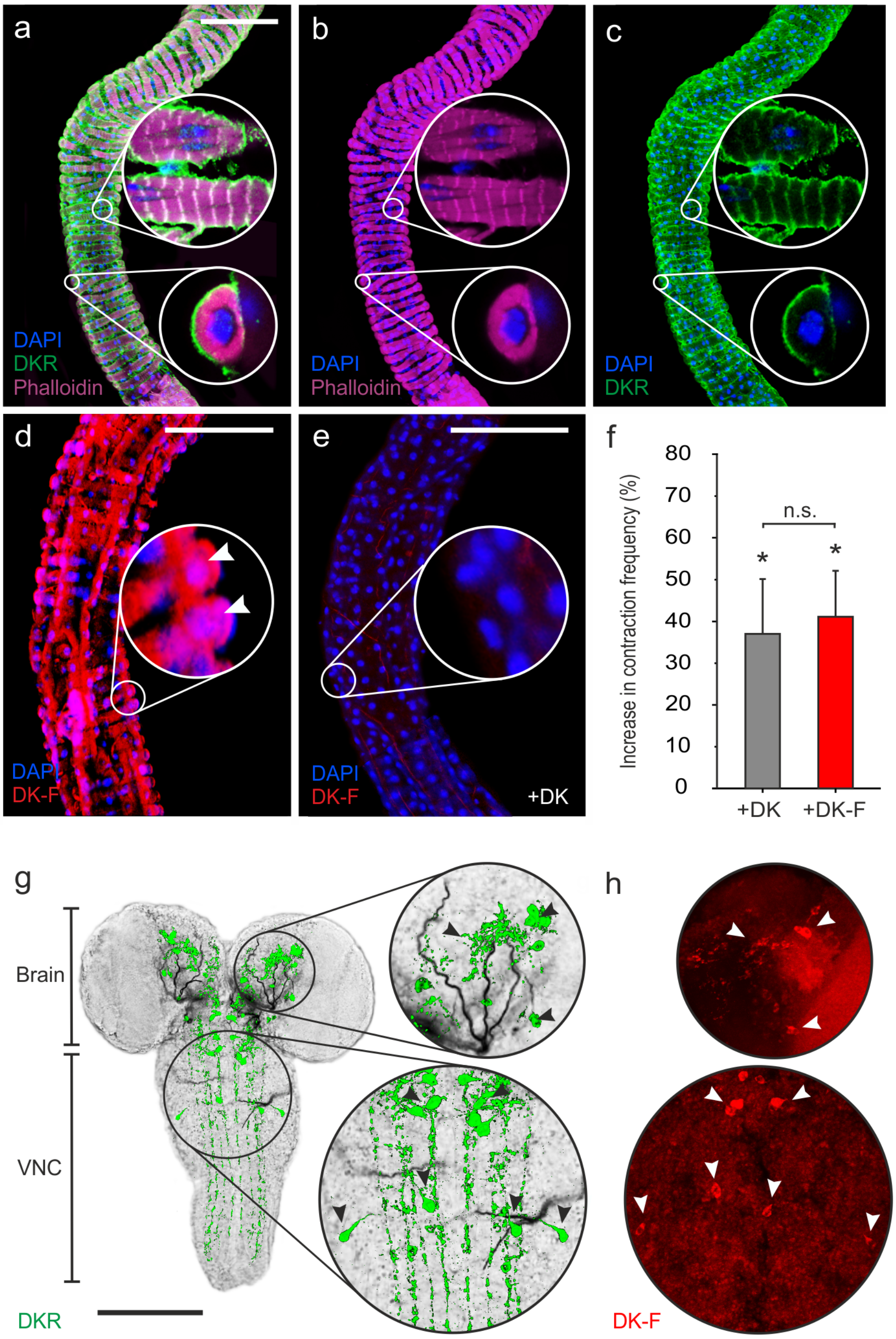

Supplementary Figure 2 | DK-F reports DKR localization in hindgut and CNS of *Drosophila*. (**a-c**) Immunolocalization of DKR suggested that the receptor is highly expressed on circular muscle cells, but not epithelial cells, of the adult *Drosophila* hindgut. Maximum projection of confocal z-series with inserts showing single focal plane images of selected regions. (**d**) Application of DK-F (10^{-7} M) confirmed that the receptor localized to the surface off circular muscle cells of the adult hindgut. (**e**) Competitive inhibition with the unlabeled peptide (DK, 10^{-5} M) almost fully abolished the fluorescent signal demonstrating specificity of the ligand-receptor interaction. (**d-e**) Maximum projections of confocal z-series with inserts showing magnifications of selected regions. (**f**) Myotropic action of DK-F and DK (10^{-7} M) on the hindgut of adult *Drosophila*. Both peptides significantly increased (*; $P<0.05$, paired-samples t-test) the frequency of hindgut contractions with respect to basal conditions. The increase in contraction frequency was not significantly different (n.s.; $P>0.05$, unpaired two-sample t-test) between the two treatments. Values are expressed as mean ± s.e.m. (n=6). (**g**) Immunolocalization of DKR in CNS of 3rd instar larvae. (**h**) Corresponding regions following DK-F (10^{-7} M) application showed partial staining of major regions. (**g-h**) Maximum projections of confocal z-series with inserts showing magnifications of selected regions. Scale bars: 100μm.

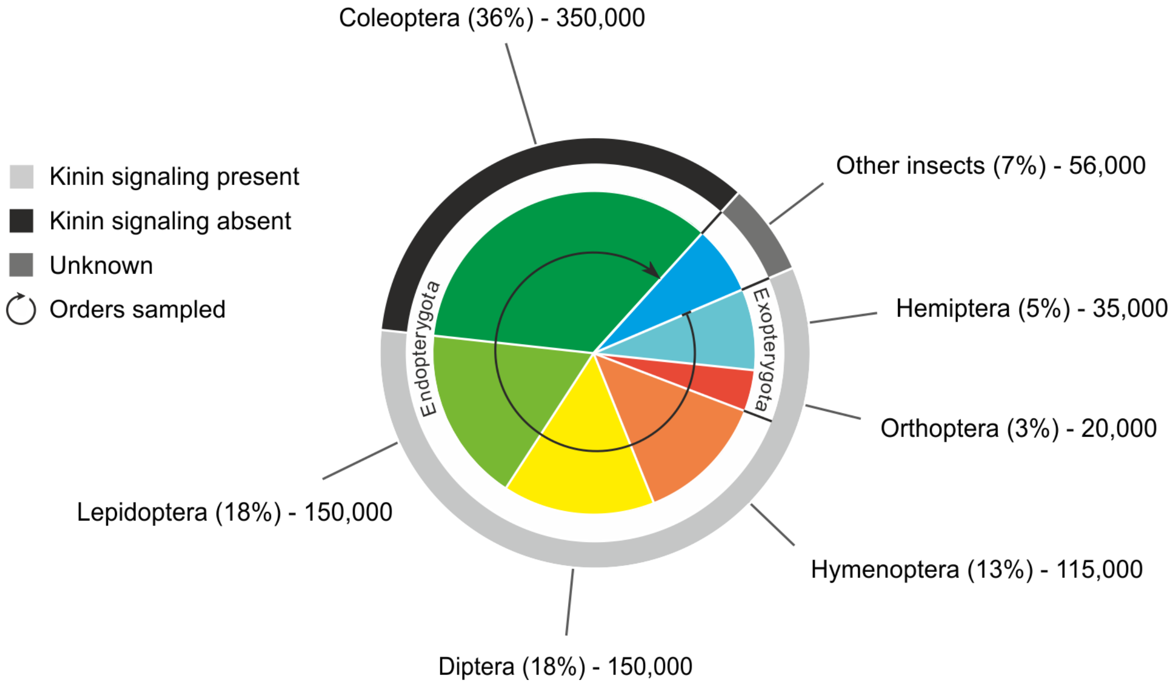

Supplementary Figure 3 | Insect biodiversity and the absence/presence of kinin signaling systems. Pie chart illustrating the relative biodiversity of each insect Order represented by our study (out of the total number of insect species described: modified from[3]), if they are grouped with the ancestral exopterygotes or derived endopterygotes, and whether they possess kinin-signaling systems. Our strategic, compact sampling thus covers approximately 93% of insect biodiversity.

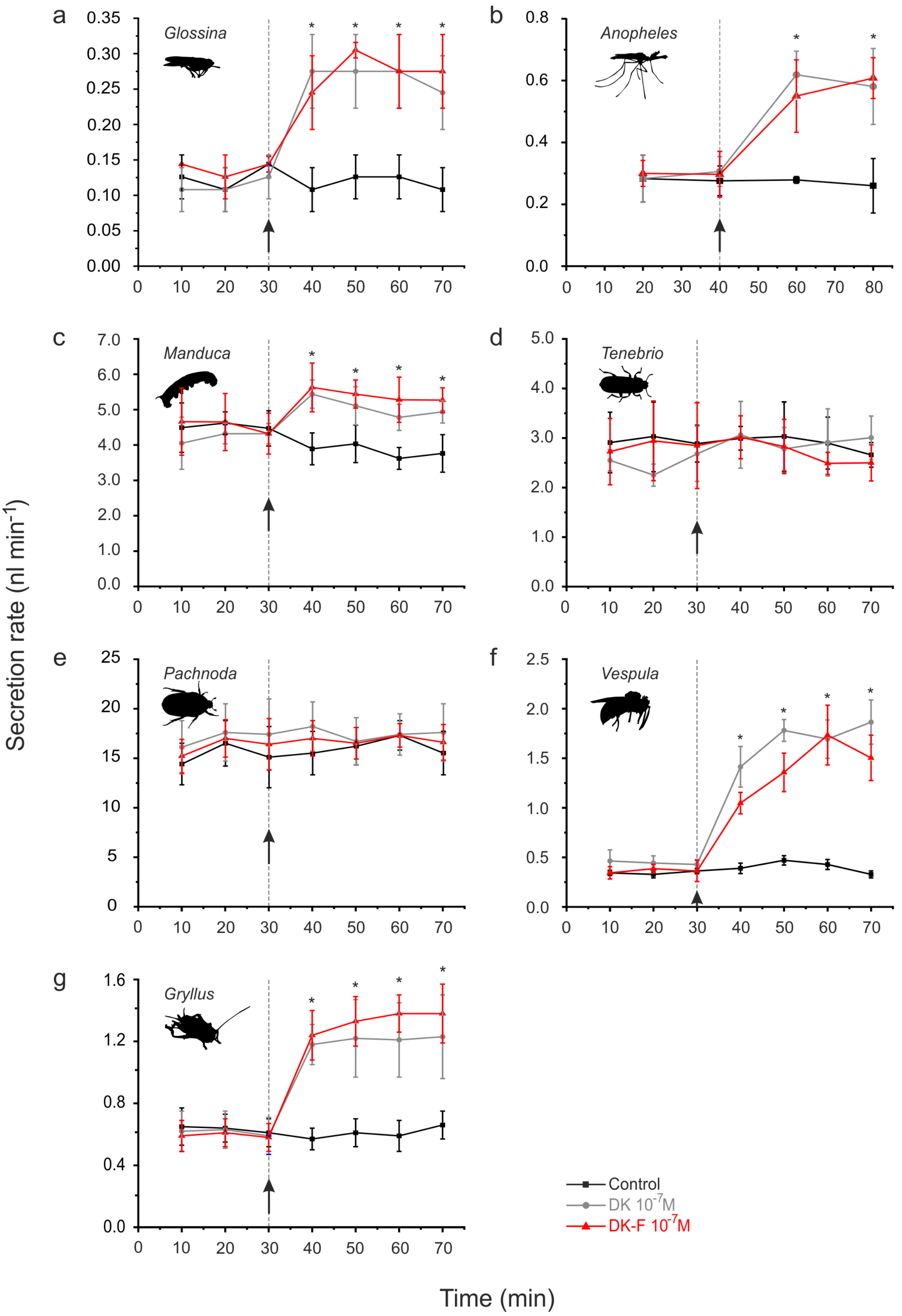

Supplementary Figure 4 | DK-F is biologically active. Fluid secretion assays with DK-F and DK on MTs from (**a**) *Glossina morsitans* (*n*=3), (**b**) *Anopheles gambiae* (*n*=4), (**c**) *Manduca sexta* (*n*=4), (**d**) *Tenebrio molitor* (*n*=4), (**e**) *Pachnoda marginata* (*n*=4) (**f**) *Vespula vulgaris* (*n*=6) and (**g**) *Gryllus assimilis* (*n*=6). Black arrow indicates time of peptide application. Values are expressed as mean ± s.e.m. Significant difference from basal rates of fluid secretion (i.e. immediately prior to peptide application) was tested using a paired samples *t*-test with a significance level of $P < 0.05$ (*).

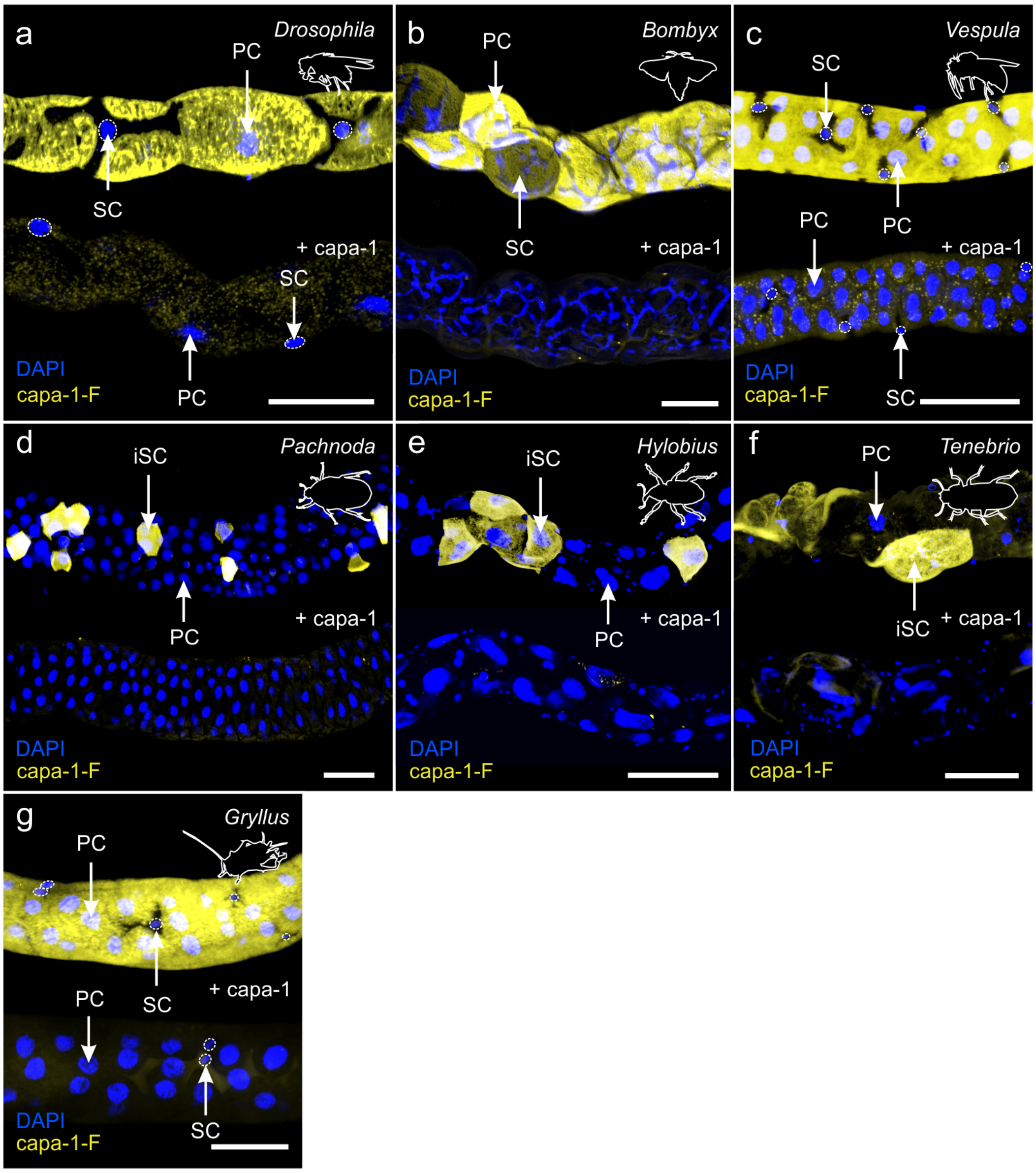

Supplementary Figure 5 | capa-1-F maps capa receptor localization. Application of capa-1-F (10^{-7} M) maps capa receptors to a PC type in endopterygote insects, such as (**a**) *Drosophila melanogaster* (Diptera), (**b**) *Bombyx mori* (Lepidoptera) and (**c**) *Vespula vulgaris* (Hymenoptera). However, capa-1-reactivity was localized to a distinct iSC in tubules of Coleoptera, such as (**d**) *Pachnoda marginata*, (**e**) *Hylobios abietis* or (**f**) *Tenebrio molitor*. In an exopterygote (**g**) *Gryllus assimilis*, capa receptors mapped to PCs; however, the small SCs showed no reactivity to any of the neuropeptides tested. In all cases, excess unlabeled peptide (+ capa-1) reduced the fluorescent signal, thus indicating that the binding was specific. PC, principal cell; SC, secondary cell; iSC, 'inverse' secondary cell type. Scale bars, 25 µm in (**a**) and (**c**), but 50 µm in (**b**), (**d**), (**e**), (**f**) and (**g**).

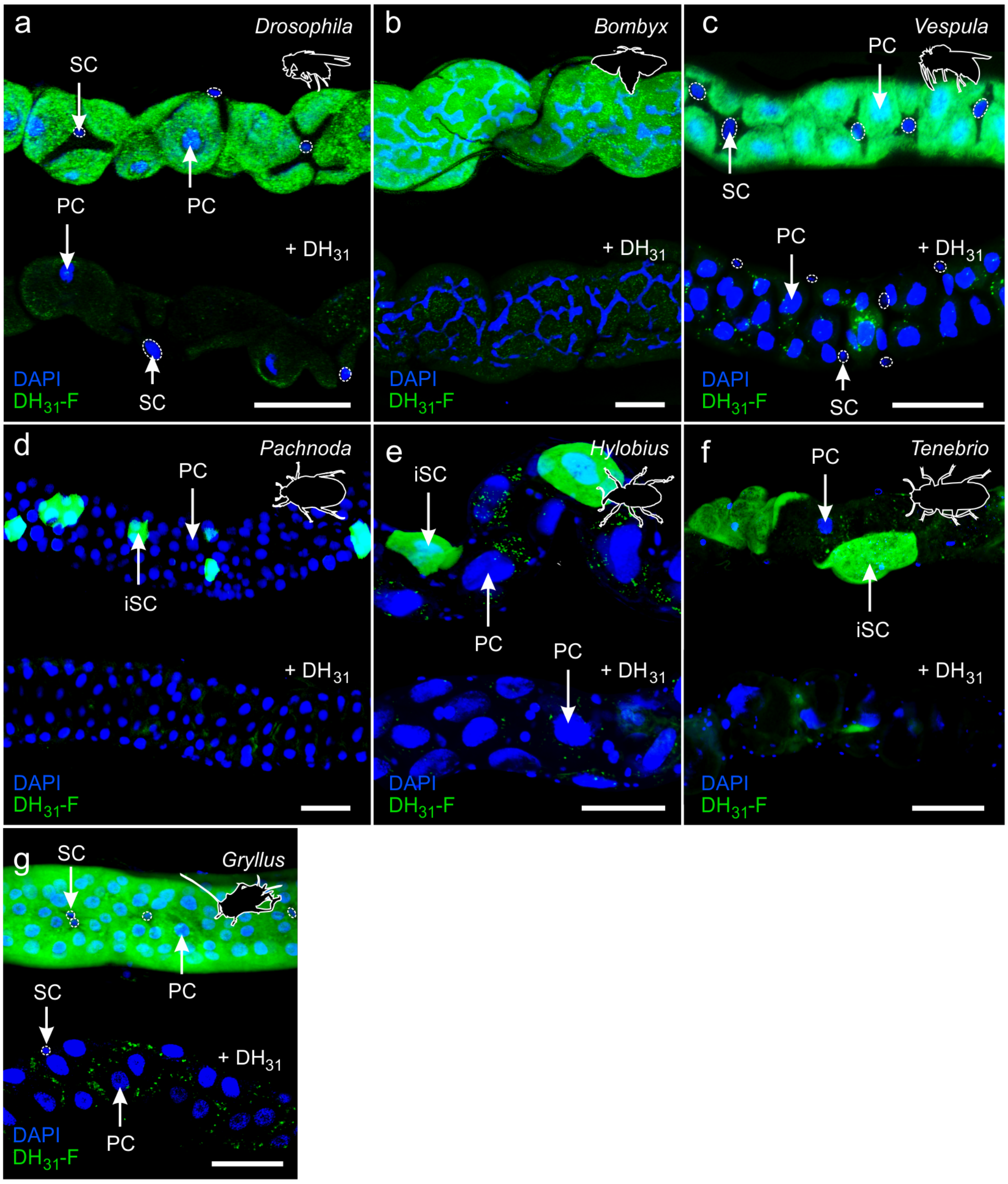

Supplementary Figure 6 | DH_{31}-F maps receptor localization. Application of DH_{31}-F (10^{-7} M) maps calcitonin-like (DH_{31}) receptors to a PC type in endopterygote insects, such as (**a**) *Drosophila melanogaster* (Diptera), (**b**) *Bombyx mori* (Lepidoptera) and (**c**) *Vespula vulgaris* (Hymenoptera). By contrast, DH_{31}-reactivity colocalized with capa receptors to a distinct cell type in tubules of Coleoptera, such as (**d**) *Pachnoda marginata*, (**e**) *Hylobios abietis* or (**f**) *Tenebrio molitor*. In an exopterygote (**g**) *Gryllus assimilis*, DH_{31} receptors mapped to PCs; however, the small SCs showed no reactivity to any of the neuropeptides tested. In all cases, excess unlabeled peptide (+ DH_{31}) reduced the fluorescent signal, thus indicating that the binding was specific. PC, principal cell; SC, secondary cell; iSC, 'inverse' secondary cell. Scale bars, 25 µm in (**a**) and (**c**), but 50 µm in (**b**), (**d**), (**e**), (**f**) and (**g**).

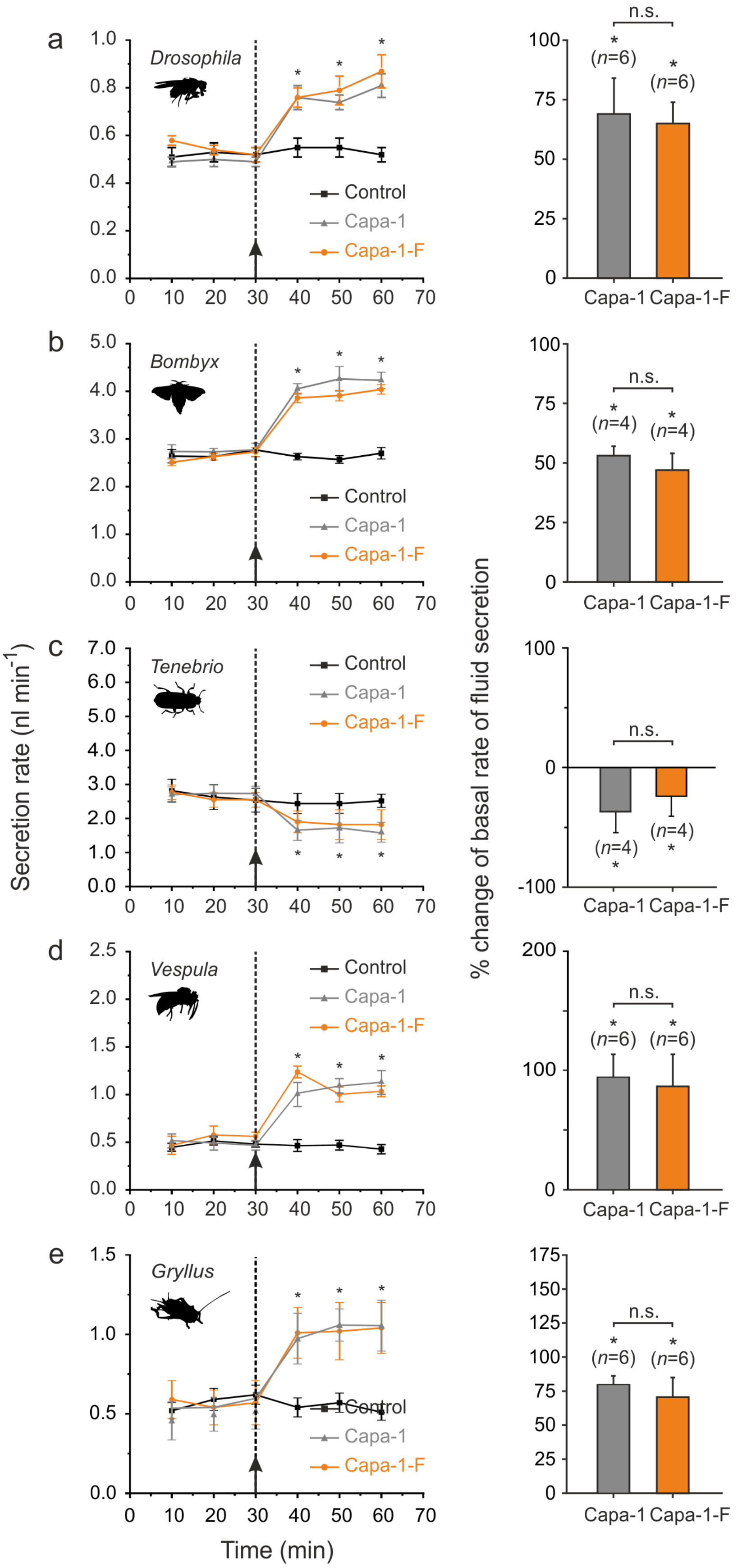

Supplementary Figure 7 | Capa-1-F is biologically active. Fluid secretion assays with capa-1-F and capa-1 on MTs from (**a**) *Drosophila melanogaster*, (**b**) *Bombyx mori,* (**c**) *Tenebrio molitor*, (**d**) *Vespula vulgaris* and (**e**) *Gryllus assimilis*. Bar graphs indicate the percent change in rate of fluid secretion immediately prior to peptide application compared to 30 min after stimulation. Black arrows indicate time of peptide application. Values are expressed as mean ± s.e.m. Significant difference from basal rates of fluid secretion was tested using a paired samples *t*-test, while an unpaired two-sample *t*-test was used to test differences in secretory response between capa-1-F and capa-1 treatments. In both cases, a significance level of $P<0.05$ (*) was taken as the critical value. n.s., not significant.

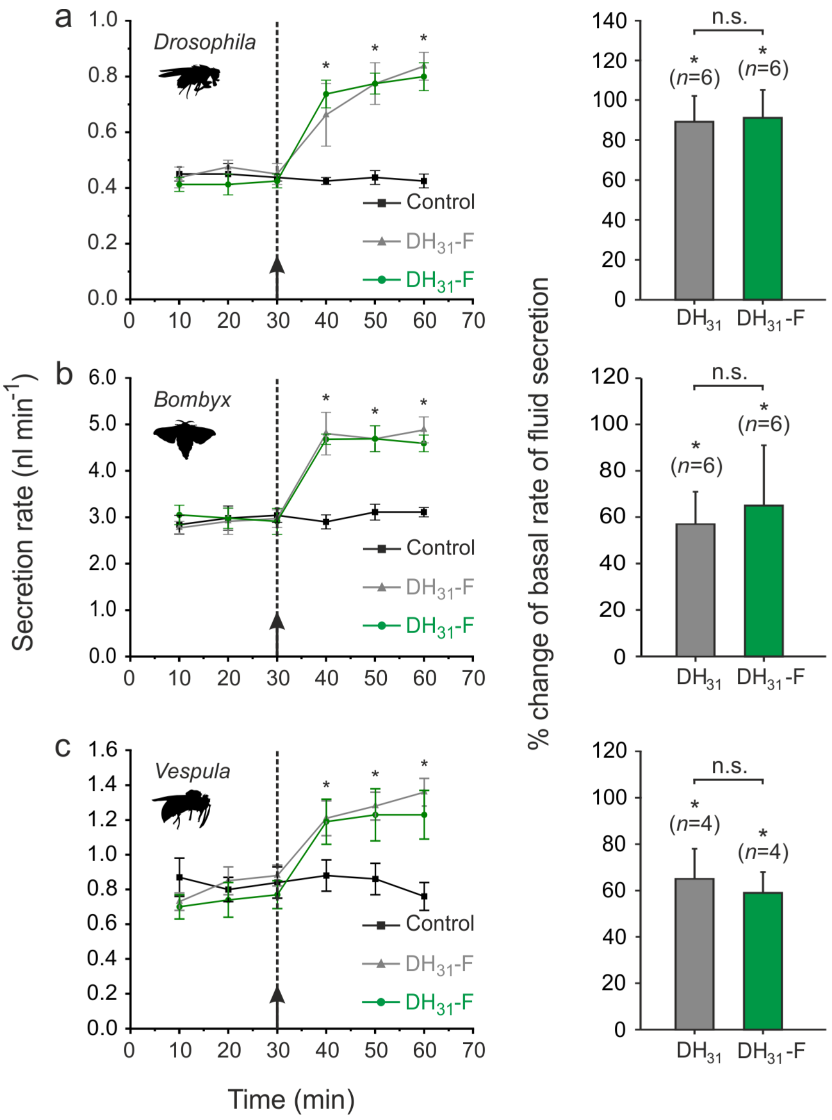

Supplementary Figure 8 | DH$_{31}$-F is biologically active. Fluid secretion assays with DH$_{31}$-F and DH$_{31}$ on MTs from (**a**) *Drosophila melanogaster*, (**b**) *Bombyx mori* and (**c**) *Vespula vulgaris*. Bar graphs indicate the percent change in rate of fluid secretion immediately prior to peptide application compared to 30 min after stimulation. Black arrows indicate time of peptide application. Values are expressed as mean ± s.e.m. Significant difference from basal rates of fluid secretion was tested using a paired samples *t*-test, while an unpaired two-sample *t*-test was used to test differences in secretory response between DH$_{31}$-F and DH$_{31}$ treatments. In both cases, a significance level of $P<0.05$ (*) was taken as the critical value. n.s., not significant.

	Species	Number	Sequence	Reference
Diptera	*Drosophila melanogaster*	I	NSVVLGKKQRFHSWGamide	4
	Glossina morsitans	-	n.a.	-
	Tipula oleracae	-	n.a.	-
	Anopheles gambiae	I	DTPRYVSKQKFHSWGamide	5
		II	NPFHSWGamide	
		III	NTAQVFYPWGamide	
Lepidoptera	*Manduca sexta*	-	n.a.	-
	Helicoverpa zea	I	YFSPWGamide	6
		II	VRFSPWGamide	
		III	KVKFSAWGamide	
	Bombyx mori	II	VRFSPWGamide	7
		III	KVKFSAWGamide	
Hymenoptera	*Vespula vulgaris*	-	n.a.	-
	Apis mellifera	I	GVFDRWGamide	8
		II	FHWIPFNSWGamide	
		III	TKFNPWGamide	
Coleoptera	*Tenebrio molitor*	-	n.a.	-
	Tribolium castaneum	-	n.p.	9
	Pachnoda marginata	-	n.a.	-
	Hylobius abietis	-	n.a.	-
Hemiptera	*Philaenus spumarius*	-	n.a.	-
	Acyrthosiphon pisum	I	QKTVFSSWGamide	10
		II	QSTYPYGamide	
		III	PAFSSWGamide	
		IV	ASDKHGamide	
		V	PKQTFSSWGamide	
		VI	SSDFFPWGamide	
Dictyoptera	*Leucophaea maderae*	I	DPAFNSWGamide	11
		II	DPGFSSWGamide	
		III	DPGFNSWGamide	12
		IV	DPGFHSWGamide	
		V	DPGFSSWGamide	13
		VI	DPGFHSWGamide	
		VII	DPAFSSWGamide	14
		VIII	GADFYSWGamide	

Supplementary Table 1 | Comparison of the amino acid sequences of the insect kinins reveals a highly conserved C-terminal pentapeptide sequence ($FX_1X_2WGamide$). Residues identical to those of DK are highlighted in black. n.a., not available; n.p., not present.

	Species	Number	Sequence	Reference
Diptera	*Drosophila Melanogaster*	I	GANMGLYAFPRVamide	15
		II	ASGLVAFPRVamide	
	Glossina morsitans	-	n.a.	-
	Anopheles gambiae	I	GPTVGLFAFPRVamide	15
		II	pQGLVPFPRVamide	
Lepidoptera	*Manduca sexta*	I	DGVLNLYPFPRVamide	15
		II	pQLYAFPRVamide	
	Bombyx mori	I	PDGVLNLYPFPRVamide	15
		II	QLYAFPRVamide	
Hymenoptera	*Vespula vulgaris*	-	n.a.	-
	Solenopsis invicta	I	SAGLVAYPRIamide	16
		II	KSDLFPRLamide	
		III	TFGIIQKPRVamide	
Coleoptera	*Tenebrio molitor*	-	n.a.	-
	Tribolium castaneum	I	NKLASVYALTPSLRVamide	9
		II	RIGKMVSFPRIamide	
Hemiptera	*Acyrthosiphon pisum*	I	ESAVAGLIPFPRVamide	17
		II	EGLIPFPRIamide	
	Nezera viridula	I	DQLFPFPRVamide	15
		II	EQLIPFPRVamide	
Dictyoptera	*Leucophaea maderae*	I	GSSGLIPFGRTamide	15
		II	GSSGLISMPRVamide	
		III	GSSGMIPFPRVamide	

Supplementary Table 2 | Comparison of the amino acid sequences of the insect capa neuropeptides reveals a conserved C-terminal tetrapeptide sequence (FPRVamide). Residues identical to those of *Drosophila* capa-1 are highlighted in black. n.a., not available.

	Species	Sequence	Reference
**Diptera**	*Drosophila Melanogaster*	TVDFGLARGYSGTQEAKHRMGLAAANFAGGPamide	[18]
	Glossina morsitans	n.a.	-
	Anopheles gambiae	TVDFGLSRGYSGAQEAKHRMAMAVANFAGGPamide	[19]
**Lepidoptera**	*Bombyx mori*	AFDLGLGRGYSGALQAKHLMGLAAANFAGGPamide	[20]
**Hymenoptera**	*Vespula vulgaris*	n.a.	-
	Apis mellifera	GLDLGLSRGFSGSQAAKHLMGLAAANYAGGPamide	[21]
	Nasonia vitripennis	GLDLGLNRGFSGSQAAKHLMGLAAANYAGGPamide	[22]
**Coleoptera**	*Tenebrio molitor*	n.a.	-
	Tribolium castaneum	GLDLGLGRGFSGSQAAKHLMGLAAANFAGGPamide	[9]
**Hemiptera**	*Acyrthosiphon pisum*	GLDLGLSRGYSGTQAAKHLMGMAAANFAGGPamide	[17]
	Rhodnius prolixus	GLDLGLSRGFSGSQAAKHLMGLAAANYAGGP amide	[23]
**Dictyoptera**	*Diploptera punctate*	GLDLGLSRGFSGSQAAKHLMGLAAANYAGGP amide	[24]

Supplementary Table 3 | Comparison of the amino acid sequences of the insect DH_{31} neuropeptides reveals an evolutionary conserved sequence. Residues identical to those of *Drosophila* DH_{31} are highlighted in black. n.a., not available.

mmol l^{-1}	*Drosophila melanogaster*	*Glossina morsitans*	*Anopheles gambiae*	*Manduca sexta*	*Tenebrio molitor*	*Vespula vulgaris*	*Gryllus assimilis*
NaCl	117.5	118	117.5	-	90	40	115
KCl	20	10	-	32	50	40	12
$MgCl_2$	2	2	8.5	1	5	5	4
$CaCl_2$	2	2	2	1	2	14	3.5
$NaHCO_3$	10.2	-	10.2	3	6	6	10
NaH_2PO_4	4.5	5	4.3	2	4	4	4
HEPES	8.6	-	8.6	-	-	-	-
Glycine	-	-	-	-	10	10	2
Proline	-	-	-	-	10	10	5
Serine	-	-	-	-	10	10	-
Histidine	-	-	-	-	10	10	1
Glutamine	-	-	-	-	10	10	2
Glucose	20	20	20	200	50	100	10
Sucrose	-	-	-	-	-	-	78
pH*	6.8	7.0	7.0	6.7	7.0	7.0	7.1
mOsm/kg**	354	300	300	320	390	390	380
Reference	[25]	[26]	[19]	[27]	[28]	[29]	[30]

Supplementary Table 4 | Experimentally optimized salines used for acute dissections and functional assays.

Supplementary References

1. Chintapalli, V. R., Wang, J. & Dow, J. A. T. Using FlyAtlas to identify better *Drosophila* models of human disease. *Nature Gen.* **39**, 715-720 (2007).

2. Robinson, S. W., Herzyk, P., Dow, J. A. T & Leader, D. P. FlyAtlas: database of gene expression in the tissues of *Drosophila melanogaster*. *Nucl. Acids Res.* **41**, D744-750 (2013).

3. Wilson, E. O. *The Diversity of Life*. Belknap Press of Harvard University Press (1992).

4. Terhzaz, S., *et al.* Isolation and characterization of a leucokinin-like peptide of *Drosophila melanogaster*. *J. Exp. Biol.* **202**, 3667-3676 (1999).

5. Radford, J. C., Terhzaz, S., Cabrero, P., Davies, S. A. & Dow, J. A. T. Functional characterisation of the *Anopheles* leucokinins and their cognate G-protein coupled receptor. *J. Exp. Biol.* **207**, 4573-4586 (2004).

6. Blackburn, M. B., Wagner, R. M., Shabanowitz, J., Kochansky, J. P., Hunt, D. F. & Raina, A. K. The isolation and identification of three diuretic kinins from the abdominal ventral nerve cord of adult *Helicoverpa zea*. *J. Insect Physiol.* **41**, 723-730 (1995).

7. Liu, X., Ning, X., Zhang, Y., Chen, W., Zhao, Z. & Zhang, Q. Peptidomic Analysis of the Brain and Corpora Cardiaca-Corpora Allata Complex in the *Bombyx mori*. *Int. J. Pept.* **2012**, 640359 (2012).

8. Veenstra, J. A., Rodriguez, L. & Weaver, R. J. Allatotropin, leucokinin and AKH in honey bees and other Hymenoptera. *Peptides* **35**, 122-130 (2012).

9. Li, B., *et al.* Genomics, transcriptomics, and peptidomics of neuropeptides and protein hormones in the red flour beetle *Tribolium castaneum*. *Gen. Res.* **18**, 113-122 (2008).

10. Huybrechts, J., *et al.* Neuropeptide and neurohormone precursors in the pea aphid, *Acyrthosiphon pisum*. *Insect Mol. Biol.* **19**, 87-95 (2010).

11. Holman, G. M., Cook, B. J. & Nachman, R. J. Isolation, primary structure and synthesis of two neuropeptides from *Leucophaea maderae*: members of a new family of cephalomyotropins. *Comp. Biochem. Physiol. C* **84**, 205-211 (1986).

12. Holman, G. M., Cook, B. J. & Nachman, R. J. Primary structure and synthesis of two additional neuropeptides from *Leucophaea maderae* - members of a new family of cephalomyotropins. *Comp. Biochem. Physiol. C* **84**, 271-276 (1986).

13. Holman, G. M., Cook, B. J. & Nachman, R. J. Isolation, primary structure, and synthesis of leucokinins V and VI: Myotropic peptides of *Leucophaea maderae*. *Comp. Biochem. Physiol. C* **88**, 27-30 (1987).

14. Holman, G. M., Cook, B. J., Nachman, R. J. Isolation, primary structure and synthesis of Leukokinin VII and Leukokinin VIII - the final members of this new family of cephalomyotropic peptides isolated from head extracts of *Leucophaea maderae*. *Comp. Biochem. Physiol. C* **88**, 31-34 (1987).

15. Predel, R. & Wegener, C. Biology of the CAPA peptides in insects. *Cell. Mol. Life Sci.* **63**, 2477-2490 (2006).

16. Choi, M. Y., Kohler, R., Vander-Meer, R. K., Neupert, S. & Predel, R. Identification and expression of capa gene in the fire ant, *Solenopsis invicta*. *PloS one* **9**, e94274 (2014).

17. Huybrechts, J., *et al.* Neuropeptide and neurohormone precursors in the pea aphid, *Acyrthosiphon pisum*. *Insect Mol. Biol.* **19**, 87-95 (2010).

18. Coast, G. M., Webster, S. G., Schegg, K. M., Tobe, S. S. & Schooley DA. The *Drosophila melanogaster* homologue of an insect calcitonin-like diuretic peptide stimulates V-ATPase activity in fruit fly Malpighian tubules. *J. Exp. Biol.* **204**, 1795-1804. (2001).

19. Coast, G. M., Garside, C. S., Webster, S. G., Schegg, K. M. & Schooley, D. A. Mosquito natriuretic peptide identified as a calcitonin-like diuretic hormone in *Anopheles gambiae* (Giles). *J. Exp. Biol.* **208**, 3281-3291 (2005).

20. Zandawala, M. Calcitonin-like diuretic hormones in insects. *Insect Biochem. Mol. Biol.* **42**, 816-825 (2012).

21. Hummon, A. B*., et al.* From the genome to the proteome: uncovering peptides in the *Apis* brain. *Science* **314**, 647-649 (2006).

22. Hauser, F., Neupert, S., Williamson, M., Predel, R., Tanaka. Y. & Grimmelikhuijzen, C. J. Genomics and peptidomics of neuropeptides and protein hormones present in the parasitic wasp *Nasonia vitripennis*. *J. Prot. Res.* **9**, 5296-5310 (2010).

23. Brugge, V. A., Schooley, D. A. & Orchard I. Amino acid sequence and biological activity of a calcitonin-like diuretic hormone (DH31) from *Rhodnius prolixus*. *J. Exp. Biol.* **211**, 382-390 (2008).

24. Furuya, K*., et al.* Cockroach diuretic hormones: characterization of a calcitonin-like peptide in insects. *PNAS U S A* **97**, 6469-6474. (2000).

25. Dow, J. A. T., Maddrell, S. H. P, Görtz, A., Skaer, N. V., Brogan, S. & Kaiser, K. The Malpighian tubules of *Drosophila melanogaster:* a novel phenotype for studies of fluid secretion and its control. *J. Exp. Biol.* **197**, 421- 428 (1994).

26. Gee, J. D. Active transport of sodium by the Malpighian tubules of the tsetse fly *Glossina morsitans J. Exp. Biol.* **64**, 357-368 (1976).

27. Dow, J. A. T & O'Donnell, M. J. Reversible alkalinization by *Manduca sexta* midgut. *J. Exp. Biol.* **150**, 247-256 (1990).

28. Wiehart, U. I., Nicolson, S. W., Eigenheer, R. A. & Schooley, D. A. Antagonistic control of fluid secretion by the Malpighian tubules of *Tenebrio molitor*: effects of diuretic and antidiuretic peptides and their second messengers. *J. Exp. Biol.* **205**, 493-501 (2002).

29. Armengaud, C., Causse, N., Ait-Oubah, J., Ginolhac, A. & Gauthier M. Functional cytochrome oxidase histochemistry in the honeybee brain. *Brain Res.* **859**, 390-393 (2000).

30. Marshall, A. T., Cooper, P., Rippon, G. D. & Patak, A. E. Ion and Fluid Secretion by Different Segments of the Malpighian Tubules of the Black Field Cricket *Teleogryllus-Oceanicus*. *J. Exp. Biol.* **177**, 1-22 (1993).
